# Biologics for psoriasis patients under 18 years of age: Real-world evidence from the Chinese psoriasis real world evidence research group

**DOI:** 10.3389/fmed.2022.1009991

**Published:** 2022-09-07

**Authors:** Yu-Xin Zheng, Li-Ran Ye, Bing-Xi Yan, Si-Qi Chen, Sui-Qing Cai, Xiao-Yong Man

**Affiliations:** Department of Dermatology, Second Affiliated Hospital, Zhejiang University School of Medicine, Hangzhou, China

**Keywords:** pediatric psoriasis, biologics, adalimumab, secukinumab, ixekizumab

## Abstract

**Background:**

Treatment for pediatric psoriasis is challenging because of the lack of real-world evidence, especially for biological therapies.

**Objectives:**

This study evaluated the efficacy and safety of biologics in children with psoriasis based on real-world evidence.

**Methods:**

Pediatric psoriasis patients aged <18 years who were treated with biologics in our hospital (2020–2022) were prospectively analyzed. Patients treated with adalimumab, secukinumab, or ixekizumab were followed up for at least 16 weeks, and 22 of 38 patients completed the 52-week observation period. Dermatologist raters were blinded to ensure the reliability of the PASI, BSA, and PGA score assessments. PASI 75 or PGA 0/1 at week 12 represented an efficient indicator.

**Results:**

Thirty-eight patients (20 males and 18 females; median age, 12.6 ± 4.1 years) were enrolled, and none were lost to follow-up. All participants were diagnosed with psoriasis, including plaque psoriasis (*n* = 36), nail psoriasis (*n* = 1), and pustular psoriasis (*n* = 1). Within 12 weeks, all patients achieved scores above PASI 75 and PGA 0/1. The average time to reach PASI 75 was 4.3 ± 2.0, 3.2 ± 1.8, and 2.4 ± 0.4 weeks in patients using adalimumab, secukinumab, and ixekizumab, respectively, and, 27.2% (3/11), 86.4% (19/22), and 75.0% (3/4) of these patients achieved PASI 100 at week 12, respectively. Moreover, 18 of 20 patients with plaque psoriasis maintained ≥PASI 75 after 52 weeks. The most commonly reported adverse effect was upper respiratory tract infection, and no severe adverse effects were reported.

**Conclusions:**

Our real-world data demonstrated the safety and effectiveness of adalimumab, secukinumab, and ixekizumab in children with psoriasis.

## Introduction

Psoriasis affects ~1% of children worldwide (1). Currently, the treatment of pediatric psoriasis is challenging because data is limited and standardized guidelines are lacking. Thus, far, five biological agents (etanercept, adalimumab, ustekinumab, secukinumab, and ixekizumab) have been approved by the European Medicines Agency (EMA) for the treatment of psoriasis in pediatric populations. Except for adalimumab, these agents have all been approved by the American FDA for the same purpose (1–3). In addition, adalimumab is recommended by the EMA as a first-line therapy for patients aged 4 years and older with severe chronic plaque psoriasis (1). According to a study conducted in Italy, adalimumab, etanercept, and ustekinumab have been approved for pediatric psoriasis; however, conventional systemic treatments have not been approved yet (4).

Although many biologics are already available on the market and have shown high effectiveness for the treatment of adults with psoriasis, real-world data on the use of biologics in children with psoriasis are scarce, especially in Asian countries. Real-world studies are important because there are numerous of patients excluded from clinical trials, such as elderly patients (5), and patients suffering from other forms than plaque psoriasis (6). The treatment experience of these patients is primarily derived from case reports and/or real life studies. Real life data also adds important information for multifailure patients (7).

## Materials and methods

### Study design and population

In this prospective study, 38 psoriasis patients under 18 years of age were treated with adalimumab (*n* = 12), secukinumab (*n* = 22), or ixekizumab (*n* = 4) for at least 16 weeks at the Second Affiliated Hospital, Zhejiang University School of Medicine, from 2020 to 2022. All the patients were diagnosed with psoriasis, including plaque psoriasis (*n* = 36), nail psoriasis (*n* = 1), and pustular psoriasis (*n* = 1). Biologics were chosen based on the National Psoriasis Foundation guidelines (1), dermatologist assessments, drug availability and prices, and patient requirements. A 1-month drug washout period was implemented before each patient was enrolled. First, psoriasis patients treated with adalimumab received an initial dose of 80 mg adalimumab, followed by 40 mg every other week. Patients weighing more than 50 kg were treated with 300 mg secukinumab subcutaneously at week 0, 1, 2, 3, and 4, followed by 300 mg every 4 weeks. The dose of secukinumab was halved to 150 mg for patients weighing between 25 and 50kg, and for patients <25 kg, the dose was 75 mg each injection. More significantly, a 3-year-old psoriasis patient treated with secukinumab was included in the research. Actually, secukinumab was recommended for use in patients over 6 years old (2). So, before the injection, the clinician emphasized that there was no case of use in children under 6 years old and repeatedly informed of the potential risks as well as benefits. Given the severity of the disease and repeated failure of topical medication, this participant underwent a thorough examination and then admintered 75 mg secukinumab at her guardians' strong desire to try this new emerging biological agent rather than other conventional systemic therapies. Patients who received ixekizumab were prescribed 160 mg (two injections of 80 mg) subcutaneously at week 0, followed by 80 mg at weeks 2, 4, 6, 8, 10, and 12; moreover, these patients were administered the same dose (80 mg) every 4 weeks.

This study was conducted in accordance with the principles of the Declaration of Helsinki and approved by the Human Research Ethics Board of the Second Affiliated Hospital, Zhejiang University School of Medicine (approval number 2020-485). Informed consent was obtained from all the participants in the study.

### Baseline assessment and follow-up

Demographic data, Psoriasis Area and Severity Index (PASI), Body Surface Area (BSA), Physician's global assessment (PGA), Nail Psoriasis Severity Index (NAPSI) scores were collected from the Chinese Psoriasis Real World Evidence Research Group (CPRWERG). The administration of biologics to all patients was carried out in accordance with the guidelines at the beginning of the treatment period, and we considered scores of at least PASI 75 or PGA 0/1 at week 12 as efficient indicators. After 12 weeks, some patients who exceeded PASI 90 and showed stable low disease activity were asked for consent and administered an extension of the dosing interval. Successful treatment was defined when the dosing interval could be extended without loss of efficacy or when the PASI score remained below 3 for at least 6 months. Thirty-eight patients were followed for at least 16 weeks, and data on adverse events, biologic agent switching, extended dosing intervals, and disease relapse were also collected.

### Data analyses and statistical methods

Quantitative variables were expressed as the mean ± standard deviation (±SD). The Mann-Whitney test was used to assess the effectiveness of biological agents over time. Statistical analyses were performed using GraphPad Prism v.9.0.0, and *p* < 0.05 indicated significant results.

## Results

### Characteristics of the patients and biologics

Demographic data and disease characteristics of the 38 recruited patients with psoriasis are listed in Table 1. The patients included 20 males and 18 females, with a median age of 12.6 years (range, 3–17 years). Among them, 36 patients were diagnosed with plaque psoriasis, 1 was diagnosed with pustular psoriasis, and 1 was diagnosed with nail psoriasis. The average age of onset was 8.6 ± 4.0 years, and the mean psoriasis duration was 4.0 ± 3.4 years. All 38 patients were followed up for at least 16 weeks, and 22 were followed up for more than 52 weeks during the observation period. Before starting biologics therapy, all patients had previously used topical drugs, four had been treated with acitretin, one with methotrexate, and one with a Chinese herb extracted from Tripterygium wilfordii. Before the study, four children had received biological treatment. Due to a lack of efficacy, two patients (No. 4 and No. 7) who were previously treated with etanercept for 20 months and 2 weeks, respectively, switched to adalimumab. Two adalimumab primary non-responsive patients (No. 13 and No. 35) were converted to secukinumab and ixekizumab, respectively. During the study, four patients who were previously treated with adalimumab were switched to secukinumab due to loss of response.

**Table 1:** Demographic data and disease features of the diagnosed children involved in the study.

**Characteristics**	**Definitions**	***N* = 38, *n* (%) or mean (SD)**
Type	Plaque psoriasis	36 (94.7%)
	Generalized pustular psoriasis	1 (2.7%)
	Nail psoriasis	1 (2.7%)
Age, years		12.6 (±4.1)
Male		20 (52.6%)
Family history		7 (18.4%)
Age of onset, years		8.6 (±4.0)
Psoriasis duration, years		4.0 (±3.4)
Baseline PASI		15.1 (±9.0)
Baseline BSA		28.2 (±23.9)
Baseline PGA		2.9 (±1.2)
Previous treatment	Topical drugs	38 (100.0%)
	Acitretin	4 (10.5%)
	Methotrexate	1 (2.6%)
	Chinese herb	1 (2.6%)
Previous biologic	None	34 (89.5%)
	1 biologic	4 (10.5%)

Data are expressed as the mean ± standard deviation or absolute number (percentage). BSA, body surface area; PASI, psoriasis area and severity index; PGA, physician's global assessment; SD, standard deviation.

### Efficacy and safety of adalimumab, secukinumab, and ixekizumab for pediatric psoriasis

**Figure 1:**
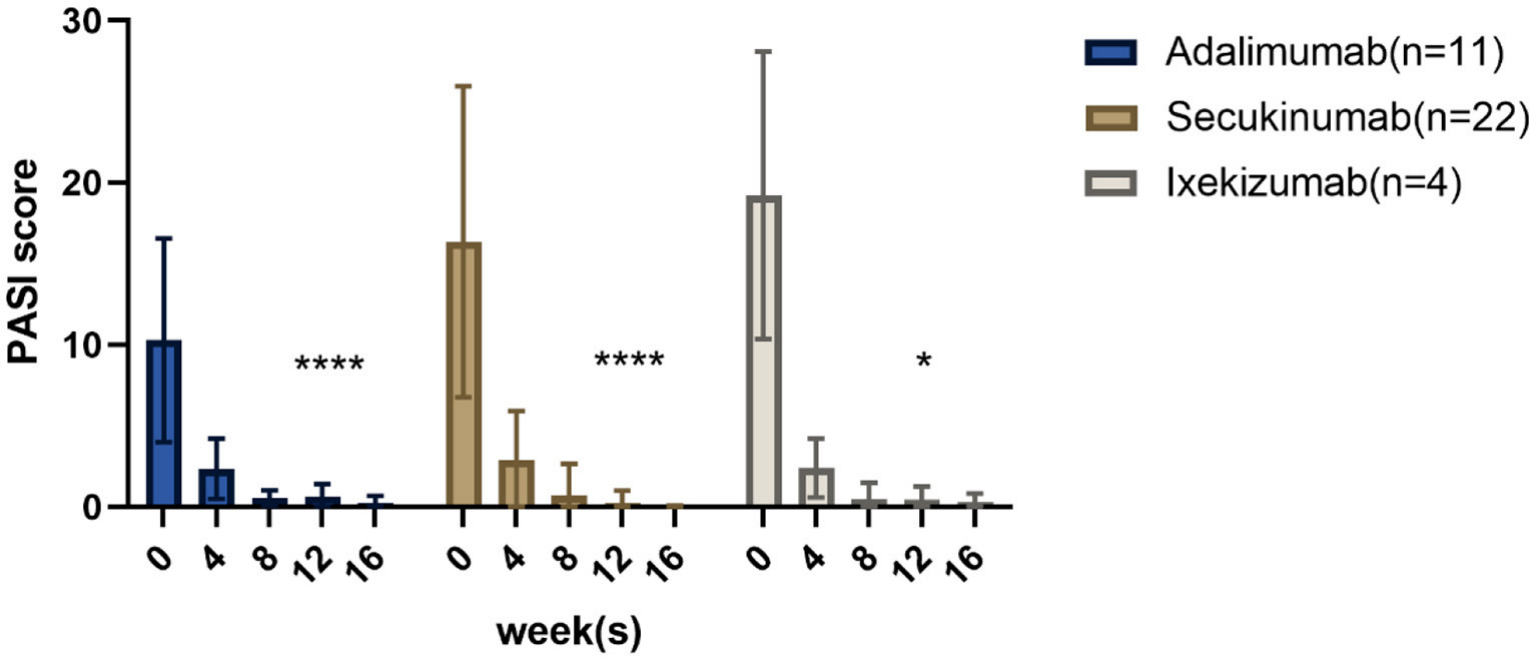
Results after 16 weeks: PASI scores of children with psoriasis treated with three different biological agents. *p < 0.05; ****p < 0.0001.

All patients in this prospective study achieved PASI ≥75 or PGA 0/1 within 12 weeks. The mean baseline BSA scores of the adalimumab, secukinumab, and ixekizumab groups decreased from 17.2, 33.6, and 29.3 to 0.7%, 0.2, and 0.5%, respectively, at 12 weeks. The changes in PASI scores from baseline to week 16 are shown in Figure 1. More detailed information on each patient is provided in Supplementary Table 1. The PASI score in the adalimumab group at week 12 was reduced significantly compared to that at baseline (*p* < 0.0001). Similar results were found in the secukinumab (*p* < 0.0001) and ixekizumab groups (*p* < 0.05). The average time to reach PASI 75 after adalimumab, secukinumab, and ixekizumab administration was 4.3 ± 2.0, 3.2 ± 1.8, and 2.4 ± 0.4 weeks, respectively. Additionally, in the fourth week, the percentage of PASI75/90/100 responders in the secukinumab group (77, 36, and 27%, respectively) was higher than that in the adalimumab group (45, 27, and 0%, respectively).

Among the 12 children treated with adalimumab (11 plaque type and 1 nail type, 7 males and 5 females, median aged 13.0 ± 3.6 years), the baseline PASI score of the 11 plaque psoriasis patients was 11.3 ± 6.9. All of these patients achieved PASI 75 at week 12, with three achieving PASI 100. In addition, the child with nail psoriasis achieved 3/4 nail growth after 12 weeks. However, 4 of the 12 children were converted to secukinumab after 6, 7, 16, and 16 months due to loss of response to adalimumab. Regarding adverse events, only two patients presented with mild upper respiratory infections during the study period.

In the secukinumab group (*n* = 22), there were 10 males and 12 females with a median age of 12.0 ± 4.5 years. The PASI score in patients receiving secukinumab at baseline was 16.4 ± 9.6. All patients achieved PASI 75 at 12 weeks, including 19 children (86.3%) who achieved PASI 100. Among the 22 patients receiving secukinumab, only two patients suffered from adverse events. One developed an upper respiratory infection and cutaneous fungal infection after the fourth injection. The other experienced pruritis.

Four patients received ixekizumab (3 males and 1 female, median aged 14.8 ± 2.9 years), and their PASI at baseline was 19.2 ± 8.9. Furthermore, after 12-week treatment, three participants reached PASI 100 while the other diagnosed with pustular psoriasis achieved PASI 90 without adverse events.

Altogether, 22 of 38 children (20 cases of plaque type psoriasis, 1 nail type, and 1 pustular type) were followed for the entire 52-week observation period. No severe disease reactivation was observed, and 18 of the 20 plaque-type patients maintained PASI ≥75. The nail-type psoriasis patient's nails all remained full length and the pustular-type patient's PASI score was valued 4.0. Adverse events of children with psoriasis are presented in Table 2.

**Table 2:** Adverse events of children with psoriasis treated with biologics.

**Biological treatment**	**Adverse events**	**Observed counts**
Adalimumab (*n* = 12)	Upper respiratory infection	2
Secukinumab (*n* = 22)	Upper respiratory infection	1
	Skin fungal infection	1
	Cutaneous pruritus	1
Ixekizumab (*n* = 4)	–	–

### Effectiveness for nail- and pustular-type psoriasis

Before administering adalimumab, the patient with nail-type psoriasis had almost no nail plate on eight fingers and two toes, which were covered with thick scales with a total NAPSI valued 48. After 12 weeks, the nails had grown to more than 3/4 of the normal length with a total NAPSI valued 11. However, from weeks 12 to 24, the patient's fingernails and toenails did not improve further. Thus, the medication was switched to secukinumab. Subsequently, the patient's nails had grown to full length and the scales almost disappeared. The patient has been using secukinumab for more than 1 year, and his nails are currently growing well-with a total NAPSI valued 1.

The patient with pustular psoriasis and a baseline PASI score of 23.0 achieved PASI 75 after 4 weeks and PASI 90 after 8 weeks. However, the plaques on the back of the neck and scalp were refractory to the treatment. At week 52, the patient was in stable condition, with a PASI value of 4.0.

### Biologics switching in seven cases

In the study, a total of 7 patients (six plaque type and one nail type) underwent biologics transitions without any washout periods, and the use of a novel biological agent led to excellent outcomes. Among them, 6 patients with plaque psoriasis obtained PASI 75 or above within 12 weeks after switching to a new biological agent (1 patient switched from etanercept to adalimumab, 3 patients switched from adalimumab to secukinumab, 1 patient switched from adalimumab to ixekizumab, 1 patient firstly switched from etanercept to adalimumab, secondly switched to secukinumab). As for the nail-type patient, all affected toenails and fingernails grew from 3/4 to full length in 8 weeks (from adalimumab to secukinumab). No severe adverse events occurred during the transition between biologics. Detailed information regarding the conversion of biologics is presented in Table 3.

**Table 3:** Biologics switching: detailed patient data.

**Patient No**.	**Biological treatment, 1st, 2nd, and 3rd Line**	**Duration**	**Reasons for biologics switching**	**≥PASI 75 response within 12 Weeks**
1	• 1st Adalimumab • 2nd Secukinumab	• 1st 7 months • 2nd 14 months	Loss of response	• 1st Yes • 2nd Yes
4	• 1st Etanercept • 2nd Adalimumab • 3rd Secukinumab	• 1st 20 months • 2nd 16 months • 3rd 14 months	Loss of response	• 1st Yes • 2nd Yes • 3rd Yes
6	• 1st Adalimumab • 2nd Secukinumab	• 1st 16 months • 2nd 8 months	Loss of response	• 1st Yes • 2nd Yes
7	• 1st Etanercept • 2nd Adalimumab	• 1st 2 weeks • 2nd 23 months	Primary non-response and deteriorated	• 1st No • 2nd Yes
12	• 1st Adalimumab • 2nd Secukinumab	• 1st 6 months • 2nd 14 months	Loss of response	• 1st 3/4 nail growth • 2nd full nail growth
13	• 1st Adalimumab • 2nd Secukinumab	• 1st 6 weeks • 2nd 13 months	Primary nonresponse	• 1st No • 2nd Yes
35	• 1st Adalimumab • 2nd Ixekizumab	• 1st 5 weeks • 2nd 20 months	Primary nonresponse	• 1st No • 2nd Yes

### Evidence on extending the dose interval and disease recurrence

Treatment with biologics was initiated according to the registered standard dose (refer to the “Study design and population” subsection in “Materials and Methods” section) and was tapered after a certain period when the patients reached PASI 90 or PASI 100. Adalimumab was tapered from a standard dose of 40 mg Q2W to 40 mg Q4W. Patients treated with secukinumab were tapered to 75 mg/150 mg/300 mg Q8W after receiving the standard dose (75 mg/150 mg/300 mg Q4W). Moreover, the ixekizumab dose was tapered from 80 mg Q4W to 80 mg Q8W. In each group, 7 of 8 (87.5%) adalimumab patients, 10 of 10 (100%) secukinumab patients, and 1 of 3 (33.3%) ixekizumab patients could successfully prolong their dosing interval maintaining PASI 90 or above and the absolute PASI score <3 for at least 6 months. Detailed information on the extended drug delivery interval is shown in Table 4.

**Table 4:** Extending the dose interval: detailed patient data.

**Patient No**.	**Biologic agent with extending dose interval**	**Maintain PASI ≥90 and absolute PASI <3 for at least 6 Months**
1	Secukinumab	Yes, maintained PASI 100
2	Adalimumab	Yes, maintained PASI 100
3	Adalimumab	Yes, maintained PASI 90
4	Secukinumab	Yes, maintained PASI 90
5	Adalimumab	Yes, maintained PASI 100
6	Adalimumab	No, transferred to secukinumab and was monitored
7	Adalimumab	Yes, maintained PASI 100
8	Adalimumab	Yes, maintained PASI 90
9	Adalimumab	Yes, maintained PASI 100
10	Adalimumab	Yes, maintained PASI 90
12	Secukinumab	Maintained full nail growth
13	Secukinumab	Yes, maintained PASI 90
14	Secukinumab	Yes, maintained PASI 100
15	Secukinumab	Yes, maintained PASI 90
16	Secukinumab	Yes, maintained PASI 90
18	Secukinumab	Yes, maintained PASI 100
20	Secukinumab	Yes, maintained PASI 100
21	Secukinumab	Yes, maintained PASI 100
35	Ixekizumab	Yes, maintained PASI 100
36	Ixekizumab	No, resumed regular dosing and was monitored
37	Ixekizumab	No, resumed regular dosing and was monitored

Patients not mentioned in this table were either on regular dosing or received extended dosing intervals of <6 months and were monitored.

## Discussion

As an efficient and safe treatment option, biological therapy has become an attractive treatment regimen for children with psoriasis (8). In line with the findings of other similar clinical trials (3, 9, 10), our real-world data fully demonstrate the safety and effectiveness of biologics in children with psoriasis.

The EMA recommends adalimumab as a first-line drug (4), and theoretically, this biological agent has fewer adverse events than traditional agents and thus requires a shorter period of laboratory monitoring (11). In a phase III trial (NCT01251614) (10), pediatric psoriasis patients who were treated with 0.8 mg/kg adalimumab achieved a higher PASI 75 response rate compared with those treated with methotrexate (*p* = 0.027). In addition, 61% of the patients reached PGA 0/1 at 16 weeks in this randomized controlled trial (RCT) study, and this rate increased to 100% in our study. In a real-world study (12) of clinical use of adalimumab in pediatric psoriasis patients in Italy (*n* = 54), 55.5 and 29.6% of patients achieved PASI 75 and PASI 90 at week 16, respectively. Our study improved these rates to 100 and 72.7%, respectively. The discrepancies between these studies may be attributed to the small sample size, different baseline demographic and disease characteristics and subjectivity of the PASI scores.

Secukinumab, a recombinant, fully human IL-17A inhibitor, has been approved for the treatment of pediatric psoriasis patients aged ≥6 years in both the USA and Europe (2). The efficacy of secukinumab in the management of pediatric plaque psoriasis was evaluated in two phase III trials (2). In a study by Bodemer et al. (13), secukinumab achieved higher PASI 75/90/100 responses at week 52 than etanercept. In our study, patients treated with secukinumab had a shorter average time to reach PASI 75 (3.2 ± 1.8 weeks) than those treated with adalimumab (4.3 ± 2.0 weeks). In addition, the percentage of patients reaching PASI 90 and PASI 100 in the secukinumab group was higher than that in the adalimumab group at the 4th week. It seems that secukinumab has a more rapid onset of action than adalimumab. However, this conclusion requires a larger sample size to be verified. Real-world studies of secukinumab treating children with plaque psoriasis are lacking, our study may fill this gap in real-world scenarios.

A phase III, randomized, double-blind, placebo-controlled study verified the efficacy and safety of ixekizumab in pediatric patients with plaque psoriasis (IXORA-PEDS) (3). Our current results are consistent with the findings of Paller et al. (3). Also, our findings are in line with a case report of pediatric psoriasis successfully and rapidly treated with ixekizumab (14). However, only four patients treated with ixekizumab were enrolled in our trial, which means that generalizable conclusions could not be drawn.

Reducing the doses of biologics while maintaining clinical effectiveness is a promising method of improving the safety and reducing the costs of therapy. However, clinical guidelines on dose tapering of biologics in pediatric psoriasis are lacking, and standard tapering eligibility criteria and tapering strategies are not available for such cases. Based on our observations and the results of other similar reports of extended dosing intervals in adults with psoriasis (15, 16), we extended the dosing intervals for some patients with stable conditions who achieved PASI 90 after obtaining informed consents. Regardless of adalimumab or secukinumab, a substantial number of patients could successfully maintain PASI 90 or even PASI 100 for more than half a year, and none suffered disease flares. Follow-up on these patients will be continued at the end of the study to gain additional insights into the long-term effects of dose reduction.

Biologic switching in the treatment of psoriasis is mainly performed due to a lack of clinical efficacy, and it is common in clinical practice. The subsequent biological agent is often administered at the next scheduled time point without a washout period (17). For adults with psoriasis who switch between TNF-α antagonists, prior inefficiency of biologics did not appear to affect the treatment outcomes for people switching from etanercept to adalimumab (17). Consistent with the results of previous studies, our study showed that patients who were administered etanercept and exhibited primary non-response and loss of response achieved PASI 75 and above within 12 weeks. In addition, a retrospective analysis (18) reported four cases of pediatric psoriasis patients who switched from etanercept to adalimumab because of efficacy issues. In terms of switching from TNF antagonists to IL-17A antagonists, a study conducted by Warren et al. (19) provided evidence of the safety and efficacy of secukinumab 300 mg in a difficult-to-treat patient population for which TNF-α inhibitor therapy was ineffective. Although adalimumab is considered a first-line biologic therapy in some cases, a significant number of patients in the adalimumab group were transitioned to a different biologic therapy because of the lack of sustained efficacy. Our research also described five successful cases switching from adalimumab to secukinumab and one switching from adalimumab to ixekizumab in children.The limitations of this study must also be addressed. First, the small sample size increased the difficulty of performing statistical comparisons of the effectiveness of different biologics. Therefore, the comparative effectiveness must be further investigated to understand how these treatment alternatives perform in a pediatric setting and to optimize treatment selection. Second, potential biases include recruitment, allocation, maintenance and measurements bias in a real-world study. Furthermore, a longer follow-up period is required to confirm our findings. In conclusion, our study provides valuable information for clinicians managing pediatric patients with psoriasis.

## Data availability statement

The original contributions presented in the study are included in the article/Supplementary material, further inquiries can be directed to the corresponding author/s.

## Ethics statement

The studies involving human participants were reviewed and approved by Human Research Ethics Board of the Second Affiliated Hospital, Zhejiang University School of Medicine. Written informed consent to participate in this study was provided by the participants' legal guardian/next of kin.

## Author contributions

X-YM, Y-XZ, and L-RY: methodology. Y-XZ, L-RY, and S-QCh: formal analysis and investigation. Y-XZ and B-XY: writing—original draft preparation. Y-XZ, L-RY, B-XY, and S-QCh: writing—review and editing. X-YM: funding acquisition. S-QCa and X-YM: supervision. All authors contributed to the article and approved the submitted version.

## Funding

This work was supported by grants from the National Natural Science Foundation of China (NSFC) (Nos. 81930089 and 82073426).

## Conflict of interest

The authors declare that the research was conducted in the absence of any commercial or financial relationships that could be construed as a potential conflict of interest.

## Publisher's note

All claims expressed in this article are solely those of the authors and do not necessarily represent those of their affiliated organizations, or those of the publisher, the editors and the reviewers. Any product that may be evaluated in this article, or claim that may be made by its manufacturer, is not guaranteed or endorsed by the publisher.

## Supplementary material

Supplementary Table 1 Demographic information and treatment information for each child with psoriasis.

## Abbreviations

PASI: Psoriasis Area and Severity Index
PASI 75: 75% reduction of the PASI
PASI 90: 90% reduction of the PASI
PASI100: 100% reduction of the PASI
NAPSI: Nail Psoriasis Severity Index
BSA: Body Surface Area
PGA: Physician's global assessment
FDA: Food and Drug Administration
EMA: European Medicines Agency
CPRWERG: The Chinese Psoriasis Real World Evidence Research Group.
