# Supplementary material for: Biologics for psoriasis patients under 18 years of age: Real-world evidence from the Chinese psoriasis real world evidence research group
Source: Front Med (Lausanne). 2022 Sep 7;9:1009991. doi: 10.3389/fmed.2022.1009991 (PMC9490006; doi:10.3389/fmed.2022.1009991)
Supplement: Supplementary Table 1 — Demographic information and treatment information for each child with psoriasis. [file Table_1.pdf]

Table S1 Demographic information and treatment information for each child with psoriasis

Patient	Type	Gender	Age	Age of Onset	Baseline PASI	Previous Treatment	Start Treatment of the Study	Adverse Events	Switching of Biologics	Reached 52 Weeks
**1**	Plaque	F	17	11	15		Adalimumab	No	Switched to Secukinumab at week 28	Yes
**2**	Plaque	M	16	13	9.8		Adalimumab	No		Yes
**3**	Plaque	F	10	7	12.6		Adalimumab	No		Yes
**4**	Plaque	M	15	12	3.2	Acitretin, Etanercept	Adalimumab	No	Switched to Secukinumab at week 64	Yes
**5**	Plaque	M	13	8	15.9		Adalimumab	Upper respiratory infection		Yes
**6**	Plaque	F	14	13	2.7	Tripterygium wilfordii	Adalimumab	No	Switched to Secukinumab at week 64	Yes
**7**	Plaque	M	12	9	15	Methotrexate, Etanercept	Adalimumab	No		Yes
**8**	Plaque	M	17	17	12		Adalimumab	No		Yes
**9**	Plaque	F	6	4	11.1		Adalimumab	No		Yes
**10**	Plaque	F	10	9	22		Adalimumab	No		Yes
**11**	Plaque	M	17	15	7.4		Adalimumab	No		No
**12**	Nail	M	9	7	48 (NAPSI)		Adalimumab	Upper respiratory infection	Switched to Secukinumab at week 24	Yes
**13**	Plaque	F	7	4	22.4	Adalimumab	Secukinumab	No		Yes
**14**	Plaque	M	17	13	18.6		Secukinumab	No		Yes
**15**	Plaque	M	16	13	24.8		Secukinumab	Upper respiratory infection, skin fungal infection		Yes
**16**	Plaque	M	15	9	35		Secukinumab	No		Yes
**17**	Plaque	M	17	3	12		Secukinumab	No		No

**18**	Plaque	M	17	8	7.2		Secukinumab	No	Yes
**19**	Plaque	F	9	5	12		Secukinumab	No	Yes
**20**	Plaque	M	16	13	23.5		Secukinumab	No	Yes
**21**	Plaque	F	8	6	25		Secukinumab	No	Yes
**22**	Plaque	M	8	7	25		Secukinumab	No	No
**23**	Plaque	F	8	5	17		Secukinumab	No	No
**24**	Plaque	F	13	3	22.5		Secukinumab	No	No
**25**	Plaque	F	10	10	8		Secukinumab	No	No
**26**	Plaque	M	17	5	7		Secukinumab	No	No
**27**	Plaque	F	5	5	2.5		Secukinumab	No	No
**28**	Plaque	M	13	5	21		Secukinumab	No	No
**29**	Plaque	F	16	12	12		Secukinumab	Cutaneous pruritus	No
**30**	Plaque	F	17	15	10		Secukinumab	No	No
**31**	Plaque	M	13	7	4.2	Acitretin	Secukinumab	No	No
**32**	Plaque	F	11	8	12		Secukinumab	No	No
**33**	Plaque	F	7	5	10.2		Secukinumab	No	No
**34**	Plaque	F	3	2	38.1		Secukinumab	No	No
**35**	Plaque	F	17	15	20	Acitretin, Adalimumab	Ixekizumab	No	Yes
**36**	Pustular	M	11	8	23		Ixekizumab	No	Yes
**37**	Plaque	M	14	4	27.2	Acitretin	Ixekizumab	No	Yes
**38**	Plaque	M	17	11	6.7		Ixekizumab	No	No
